# Cellular Growth Kinetics Distinguish a Cyclophilin Inhibitor from an HSP90 Inhibitor as a Selective Inhibitor of Hepatitis C Virus

**DOI:** 10.1371/journal.pone.0030286

**Published:** 2012-02-08

**Authors:** Rudolf K. F. Beran, Ruchi Sharma, Amoreena C. Corsa, Yang Tian, Justin Golde, Greta Lundgaard, William E. Delaney, Weidong Zhong, Andrew E. Greenstein

**Affiliations:** Biology Department, Gilead Sciences, Foster City, California, United States of America; Saint Louis University, United States of America

## Abstract

During antiviral drug discovery, it is critical to distinguish molecules that selectively interrupt viral replication from those that reduce virus replication by adversely affecting host cell viability. In this report we investigate the selectivity of inhibitors of the host chaperone proteins cyclophilin A (CypA) and heat-shock protein 90 (HSP90) which have each been reported to inhibit replication of hepatitis C virus (HCV). By comparing the toxicity of the HSP90 inhibitor, 17-(Allylamino)-17-demethoxygeldanamycin (17-AAG) to two known cytostatic compounds, colchicine and gemcitabine, we provide evidence that 17-AAG exerts its antiviral effects indirectly through slowing cell growth. In contrast, a cyclophilin inhibitor, cyclosporin A (CsA), exhibited selective antiviral activity without slowing cell proliferation. Furthermore, we observed that 17-AAG had little antiviral effect in a non-dividing cell-culture model of HCV replication, while CsA reduced HCV titer by more than two orders of magnitude in the same model. The assays we describe here are useful for discriminating selective antivirals from compounds that indirectly affect virus replication by reducing host cell viability or slowing cell growth.

## Introduction

Intensive efforts are underway to develop new therapies for hepatitis C virus (HCV). HCV drugs can be broadly classified into two groups by target: (1) direct acting antivirals (those that inhibit the virus by directly interacting with viral proteins) or (2) host-targeted antivirals (drugs that indirectly inhibit the virus by modulating host protein function). Treatment with ribavirin and pegylated interferon-α is the current standard of care for chronic HCV-infection. Pegylated interferon-α is a host-targeted antiviral that exerts an antiviral effect indirectly through the host immune response [1]. Multiple direct-acting antivirals have been shown to reduce viral load in patients, but rapid emergence of drug resistance is a common outcome after monotherapy [2]. Host-factor inhibitors generally offer superior barriers to resistance as compared to direct acting antivirals [3], [4], with a few notable exceptions [5]. Targeting a host factor; however, potentially introduces a higher risk of side-effects, depending on the function and nature of the host protein being targeted. Assessing the risks and benefits of unique antiviral targets remains a challenge in antiviral drug discovery.

Inhibitors of two host factor targets, Cyclophilin A (CypA) and heat-shock protein 90 (HSP90), have shown selective antiviral activity with a high barrier to resistance. The cyclosporin A-analog Debio-025, which inhibits CypA, reduced viral load in combination with pegylyated interferon-α and ribavirin, without the emergence of viral resistance and has been generally well-tolerated [4]. 17-(Allylamino)-17-demethoxygeldanamycin, (17-AAG), an HSP90 inhibitor, showed potent pre-clinical efficacy against a number of viral targets including hepatitis C [6], [7], ebola [8], hepatitis B [9], [10],and influenza [11]. Drug-resistance is suppressed by HSP90 inhibitors in polio-infected mice [3]. Further, clinical trials of 17-AAG demonstrated safety and tolerability [12], [13]. Together, these data suggest that HCV inhibitors targeting CypA or HSP90 have the potential to be well tolerated with a high barrier to resistance.

CypA and HSP90 are both chaperone proteins thought to aid in HCV replication. CypA is an 18 kDa protein that exhibits peptide-prolyl isomerase activity against a broad range of substrates [14]. HSP90 is an ATP-dependent chaperone protein [15]. Both proteins are highly abundant and play an important role in host protein folding. Both CypA and HSP90 have been shown to specifically interact with the HCV NS5A protein [16], [17]. Thus, disruption of the interaction with NS5A may be responsible for the observed antiviral activity of CsA or 17-AAG, rather than some non-specific interference with cellular processes.

Demonstrating that antiviral activity is independent of cellular toxicity is critical in antiviral drug discovery. The cell-based assays typically used in pre-clinical antiviral research generally rely on rapidly-dividing immortalized cells as hosts for viral replication [18]. In contrast, most viruses infect non-dividing cells *in vivo*. Molecules that slow immortalized-cell growth may cause an apparent (but non-specific) reduction in viral replication [19]. To further complicate interpretation of toxicity measurements in cultured cell lines, various end-point assays (*e.g.*, tetrazolium salts, Calcein AM, and luciferase-coupled assays) are often employed interchangeably despite measuring distinct surrogate markers for cell health or viability (mitochondrial reduction potential, intracellular esterase activity, and intracellular ATP concentrations, respectively). This creates the possibility that a putative antiviral molecule may exert its effect indirectly by inducing a partial or unobserved cellular toxicity (*i.e.*, a cytostatic effect). The magnitude and potency of any effect on cell growth, health, viability, and toxicity must be assessed to distinguish truly selective antiviral activity.

Here we provide *in vitro* evidence that CsA (but not 17-AAG) is a selective inhibitor of HCV replication. We compared the antiviral efficacy and toxicity of the HSP90 inhibitor 17-AAG to the CypA inhibitor CsA. Neither 17-AAG nor CsA were cytotoxic (as assessed by intracellular esterase levels) at their effective antiviral concentrations. However, when cellular growth was measured directly (by time-lapse microscopy), we observed that 17-AAG (but not CsA) slowed cellular replication at the same concentrations required to inhibit HCV replication. This suggested that 17-AAG might inhibit HCV replication by non-specifically slowing cellular growth. To further investigate this, we tested both compounds in antiviral assays with either dividing or non-dividing cells. CsA maintained antiviral activity in both rapidly-dividing and non-dividing cells, but 17-AAG did not exhibit antiviral activity in non-dividing cells. We show that 17-AAG inhibits HCV replication through slowing cellular replication, while CsA has a specific antiviral effect. Furthermore, our work describes specific assays to distinguish between compounds that selectively inhibit viral replication from those which indirectly inhibit viral replication by slowing cellular growth.

## Results

### 17-AAG potently inhibits HCV replication, but also partially reduces intracellular esterase levels

We compared the anti-HCV activity and toxicity of CsA and 17-AAG to a panel of selective antiviral (HCV-796), cytostatic (gemcitabine and colchicine), and highly toxic (Puromycin) compounds. Using a stable cell line replicating a luciferase-encoding HCV replicon, we measured viral replication levels (Renilla luciferase) across a range of drug concentrations (2 nM to 44.4 µM) three days post drug addition. In parallel, we measured intracellular esterase activity as a surrogate for cell viability using the Calcein AM reagent.

**Figure 1:**
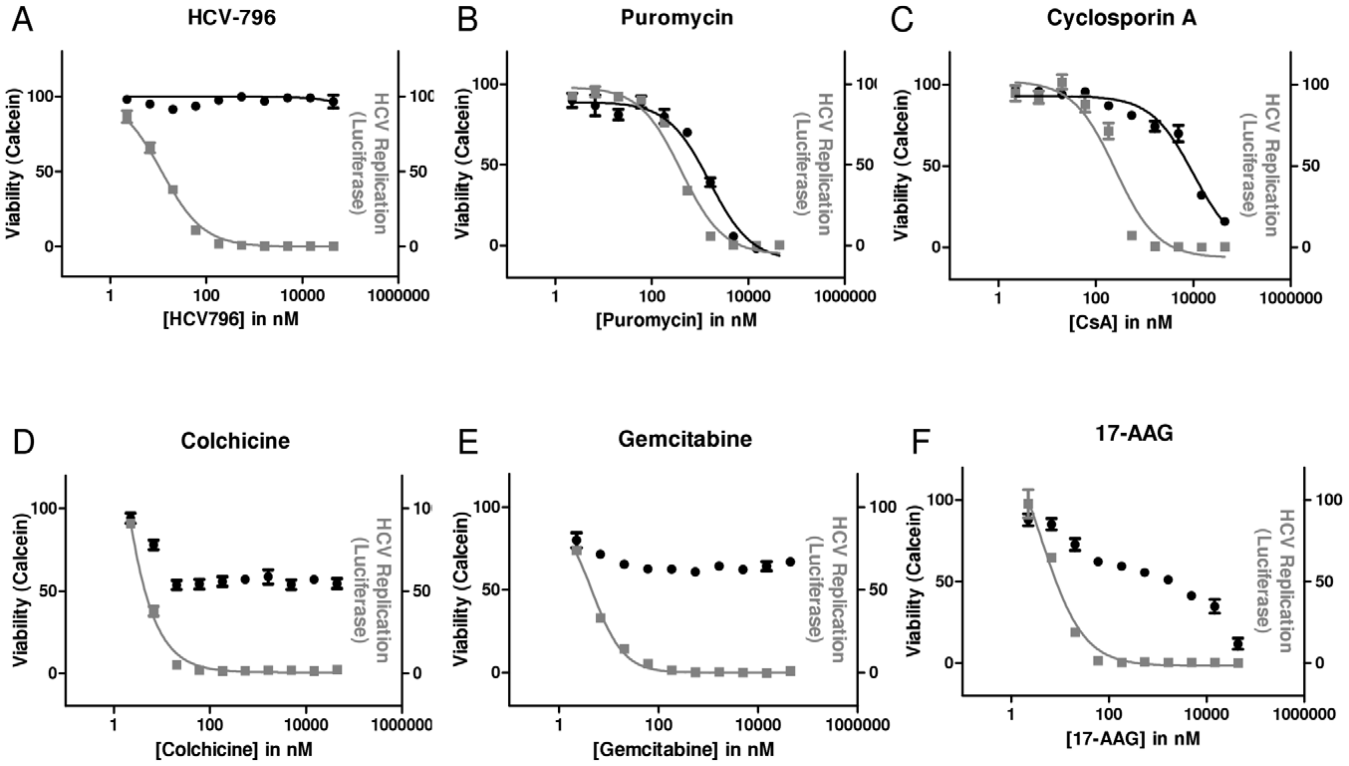
Effect of 17-AAG and CsA on HCV replication and viability determined by intracellular esterase activity. Antiviral activity (measured using the Renilla luciferase encoded by the HCV replicon; gray) and cell viability (measuring through the cleavage of calcein-AM by intracellular esterases; black) were assessed as a function of dose in a three day assay for the following molecules: (A) the HCV polymerase inhibitor HCV-796, (B) the ribosomal inhibitor puromycin, (C) the cyclophilin inhibitor CsA, (D) the microtubule inhibitor colchicine, (E) the anti-metabolite gemcitabine, and (F) the HSP90 inhibitor 17-AAG.

Antiviral replicon assays yielded standard dose-response curves ranging from 0 to 100% inhibition with EC_50_ values between 9 and 290 nM for all compounds. In contrast, the intracellular esterase activity assays yielded either no dose response (no toxicity), a full dose response (0 to 100% cell viability), or a partial dose response (*e.g.* cell viability was reduced, but reached a plateau between 0 and 50%). The HCV NS5B inhibitor HCV-796, a potent direct-acting antiviral [20], showed full antiviral activity without any observable cytotoxicity (Fig. 1A). The ribosome inhibitor puromycin (Fig. 1B), a known toxin [21], reduced intracellular esterase activity from 100% to 0% of the untreated control and fit a sigmoidal dose-response relationship. There was little difference between the apparent antiviral activity and cytotoxicity of puromycin in this assay (EC_50_ within 4-fold of CC_50_). The Cyclophilin inhibitor CsA [22] did exhibit cellular toxicity, but the concentrations required for the toxic effect were much higher (>20-fold) than the concentrations required for the antiviral effect (Fig. 1C). The microtubule inhibitor colchicine [23] (Fig. 1D) and the anti-metabolite gemcitabine [19](Fig. 1E), known cytostatic molecules, each demonstrated a full antiviral response with EC_50_ values of 9 and 12 nM, respectively (Table 1). However these compounds only partially reduced intracellular esterase activity at the majority of concentrations tested. The HSP90 inhibitor 17-AAG (Fig. 1F) demonstrated a full antiviral dose response with an EC_50_ of 12 nM (Table 1), but it had a complex, and biphasic effect on intracellular esterase activity. A partial reduction in intracellular esterase activity, similar to the colchicine and gemcitabine was observed at concentrations between 20 and 5,000 nM (Fig. 1D and E). While a CC_50_ value could be calculated in all cases (Table 1), the non-standard dose-response relationships observed may obscure the interpretation of these values. Colchicine, gemcitabine, and 17-AAG all interrupted viral replication with EC_50_ values between 9–12 nM (Table 1), but it appeared that all three compounds were also altering cellular viability at concentrations between 20 and 5,000 nM.

**Table 1:** 

Compound	Target	EC50 (nM)	CC_50_ (nM)		
			Calcein	Intracellular ATP	Cell #
HCV-796	HCV NS5B	15	>44,444	>44,444	>44,444
Cyclosporine A	Cyclophilin A	259	13,830	11,930	10,210
17-AAG	HSP90	12	21,040*	38	5
Gemcitabine	Ribonucleotide reductase	12	>44,444	6	4
Colchicine	Microtubules	9	>44,444*	16	ND**
Puromycin	Ribosome	290	1,070	516	52

*Non-standard dose-response curve.

**Altered cell morphology interferes with accurate cell number determination.

### 17-AAG potently reduces intracellular ATP concentration

**Figure 2:**
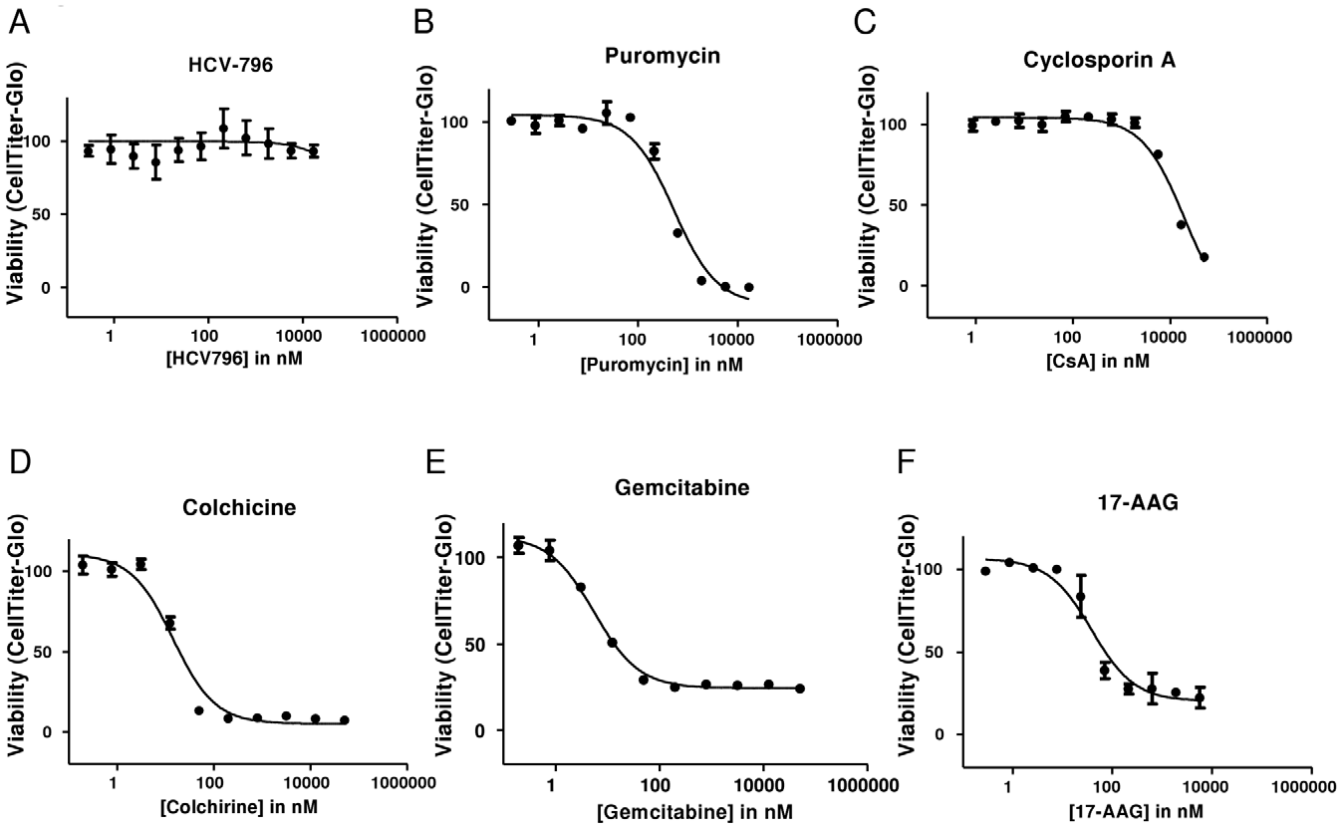
Effect of 17-AAG and CsA on cellular viability determined by measuring intracellular ATP concentration. Cell viability (measured by determining intracellular ATP levels) was assessed as a function of dose in a three day assay for the following molecules: (A) the HCV polymerase inhibitor HCV-796, (B) the ribosomal inhibitor puromycin, (C) the cyclophilin inhibitor CsA, (D) the microtubule inhibitor colchicine, (E) the anti-metabolite gemcitabine, and (F) the HSP90 inhibitor 17-AAG.

To better assess the toxicity of the host-factor inhibitors CsA and 17-AAG, we employed an alternative assay for cellular viability that measured intracellular ATP levels (CellTiter-Glo) in the same replicon cell lines. For HCV-796 (Fig. 2A), puromycin (Fig. 2B), and CsA (Fig. 2C), the intracellular ATP toxicity assay yielded results nearly identical to the Calcein assay. In contrast to the partial effect on intracellular esterase activity observed using the Calcein assay, colchicine reduced intracellular ATP signal to <7% of the untreated controls (Fig. 2D). Gemcitabine (Fig. 2E) and 17-AAG (Fig. 2F) also appeared to have potent toxicity based on the intracellular ATP assay, although the highest doses still did not fully reduce the signal to 0% of the untreated controls. Like the known-cytostatic gemcitabine, 17-AAG partially reduced viability at concentrations close to the EC_50_ value in both surrogate assays for cellular viability. In contrast, CsA, exhibited antiviral activity at concentrations 20- to 50-fold below those required for toxicity (Table 1).

### 17-AAG potently reduces cell number

**Figure 3:**
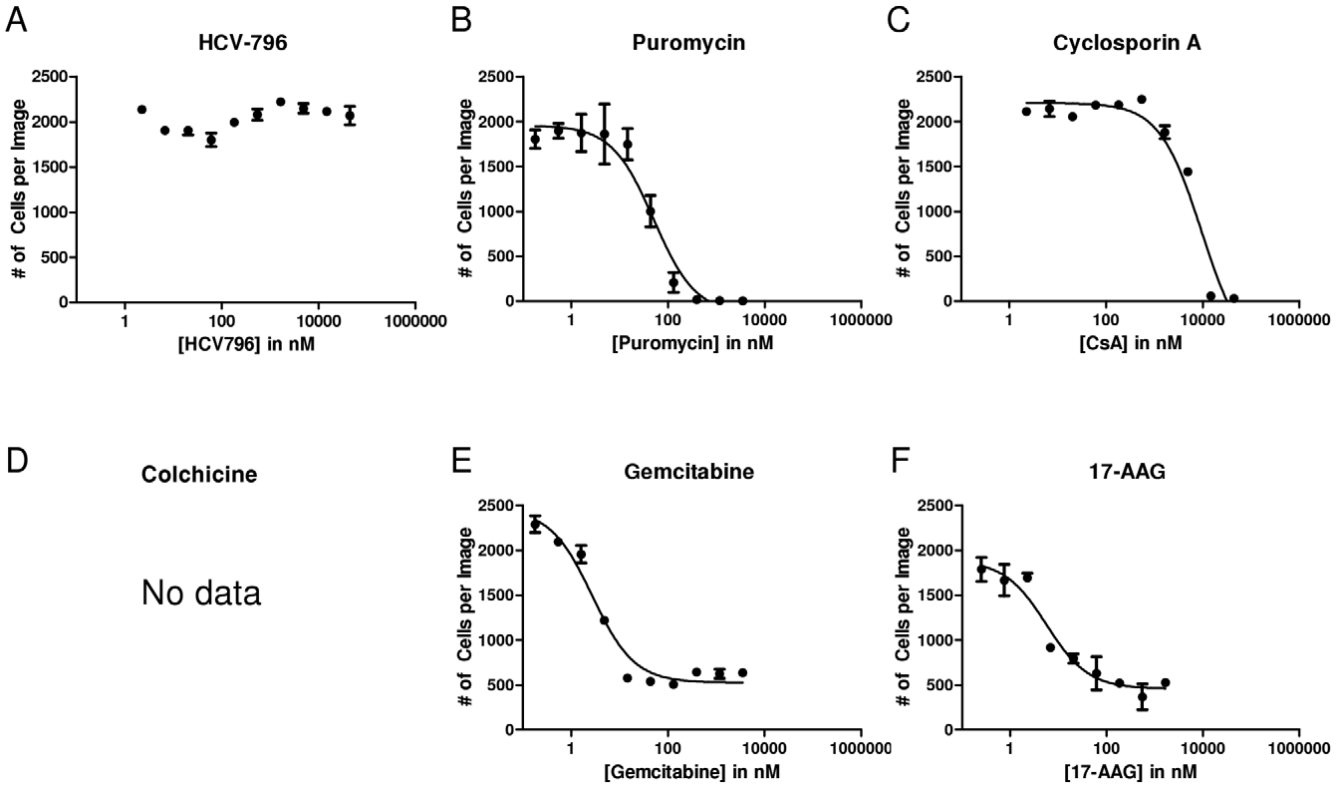
Effect of 17-AAG and CsA on cellular viability determined by cell count. Cell viability (measured by direct microscopic quantification of Hoechst-stained nuclei) was assessed as a function of dose in a three day assay for the following molecules: (A) the HCV polymerase inhibitor HCV-796, (B) the ribosomal inhibitor puromycin, (C) the cyclophilin inhibitor CsA, (D) the microtubule inhibitor colchicine, (E) the anti-metabolite gemcitabine, and (F) the HSP90 inhibitor 17-AAG. Colchicine dramatically altered cellular morphology, preventing an accurate cell count.

Because toxicity assays using different surrogates of cell number provided varying results, we next measured cell number directly by determining the number of Hoechst-stained nuclei after three days of incubation with each drug. Colchicine was excluded from this analysis because its effect on microtubules distorts cellular morphology and prevents accurate quantification of nuclei. By directly counting cells, we observed dose responses that were generally similar to those observed with the intracellular ATP assay (Fig. 3). Notably, the magnitude of the effect on cell number at the highest concentrations of compound tested was similar to the magnitude of the effect on intracellular ATP levels. HCV-796 was non-toxic at all concentrations tested. Puromycin and very high concentrations of CsA eliminated all cells in the field. Gemcitabine and 17-AAG reduced the number of cells at high concentrations but did not eliminate all cells. This observation is consistent with a cytostatic, but not cytotoxic, effect. The CC_50_ values calculated from this experiment (Table 1) were close to, but generally lower than, the CC_50_'s calculated from the intracellular ATP assay data.

**Figure 4:**
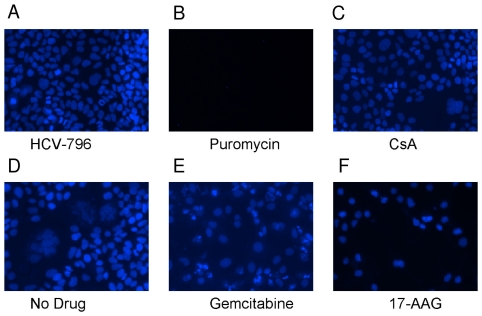
Visual assessment of replicon cell number and morphology after 1 µM compound treatment. Hoechst-stained nuclei were visualized after treatment for three days with the following molecules: (A) the HCV polymerase inhibitor HCV-796, (B) the ribosomal inhibitor puromycin, (C) the cyclophilin inhibitor CsA, (D) the microtubule inhibitor colchicine, (E) the anti-metabolite gemcitabine, and (F) the HSP90 inhibitor 17-AAG.

To better understand the effects of each compound on cell number and morphology, images of cells treated with 1 µM compound were visually inspected (Fig. 4). 1 µM puromycin eliminated all cells in the course of the three-day assay (Fig. 4B). 1 µM 17-AAG (Fig. 4F) or gemcitabine (Fig. 4E) reduced the number of cells per image to ∼22% of the controls. Neither 1 µM CsA (Fig. 4C) nor HCV-796 (Fig. 4A) altered the number of cells per image when compared to the no-drug control (Fig. 4D). In agreement with the intracellular ATP assay data, these data suggested that the antiviral effect of puromycin, gemcitabine, and 17-AAG was a consequence of adverse effects on cell health or proliferation. In contrast, CsA and HCV-796 display antiviral activity at concentrations that do not alter cell number.

### 17-AAG slows cell growth at concentrations similar to its effective antiviral concentration

**Figure 5:**
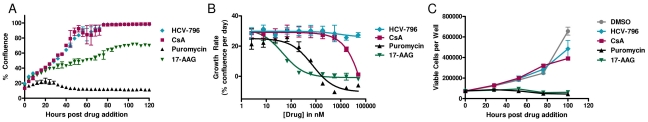
17-AAG slows cellular growth at its effective antiviral concentration. (A) Cellular growth was determined by measuring the percent confluence (by microscopic quantification) every 4 hours. HCV-796 (blue diamonds), CsA (pink squares), puromycin (black triangles), 17-AAG (green triangles). (B) Growth rate (% confluence per day) as a function of compound dose. HCV-796 (blue diamonds), CsA (pink squares), puromycin (black triangles), 17-AAG (green triangles). (C) Trypan-blue exclusion was used to determine the number of viable cells each day post drug addition. HCV-796 (blue diamonds), CsA (pink squares), puromycin (black triangles), 17-AAG (green triangles), or an equivalent volume of DMSO (grey circles).

Two possible mechanisms could cause the reduction in both intracellular ATP levels and the number of cells observed at 17-AAG concentrations close to the EC_50_: either reduced cellular proliferation rate or delayed toxicity, in which cells proliferate normally for 1–2 days, but subsequently undergo rapid cell death on days 2–3. To distinguish between these two mechanisms, we directly measured cellular confluence (the percent of the culture well occupied by cells, % confluence) every four hours and quantified cellular replication rates. 1 µM 17-AAG slowed the kinetics of cellular proliferation significantly compared to 1 µM HCV-796 (Fig. 5A). 1 µM CsA, in contrast, did not impair cellular replication rates (Fig. 5A). As a control, we confirmed the rapid elimination of cells observed with puromycin (Fig. 5A). Consistent with Figure 4, inspection of the images confirmed a decreased number of cells per field (and not an aberration in cell size or spreading) for the 17-AAG-treated cells. These data demonstrate that 1 µM 17-AAG reduced the cellular growth rate.

We next determined if 17-AAG slows cell growth at the same concentration at which it inhibits HCV replication. We quantified growth rate by assessing the change in % confluence between days 1 and 2 (% confluence/day) and measured the effect of growth rate as a function of dose. At concentrations as low as 22 nM, 17-AAG caused a significant decrease in growth rate (18.0+/−1.1% confluence/day) (Fig. 5B) as compared to the control (24.3+/−2.1% confluence/day) (Fig. 5B). This 22 nM minimum inhibitory concentration (MIC) for 17-AAG is similar to its effective concentration for inhibiting HCV replication (EC_50_ = 12 nM). Like 17-AAG, the known toxin puromycin had an MIC of 205 nM (Fig. 5B) which is also similar to its effective concentration (EC_50_ = 290 nM). HCV-796 did not significantly impair cell proliferation at any concentration tested. For CsA, the MIC was 5.6 µM (Fig. 5B) which was more than 20-fold above the EC_50_. Inhibition of CypA or HSP90; therefore, can cause very distinct effects on cellular proliferation.

Because measurements of cellular confluence do not necessarily account for cell viability, we used trypan-blue exclusion to determine the number of viable cells. Cells treated were trypsinized and quantified at multiple timepoints post drug addition (Fig. 5C). 1 µM 17-AAG or puromcyin reduced the number of viable cells equally effectively. Neither CsA nor HCV-796 treatment affected cell viability. In agreement with the intracellular ATP concentration assay data, these results suggest that 17-AAG and puromycin adversely affect cell viability at a concentration of 1 µM.

### 17-AAG does not inhibit HCV replication in non-dividing cells

**Figure 6:**
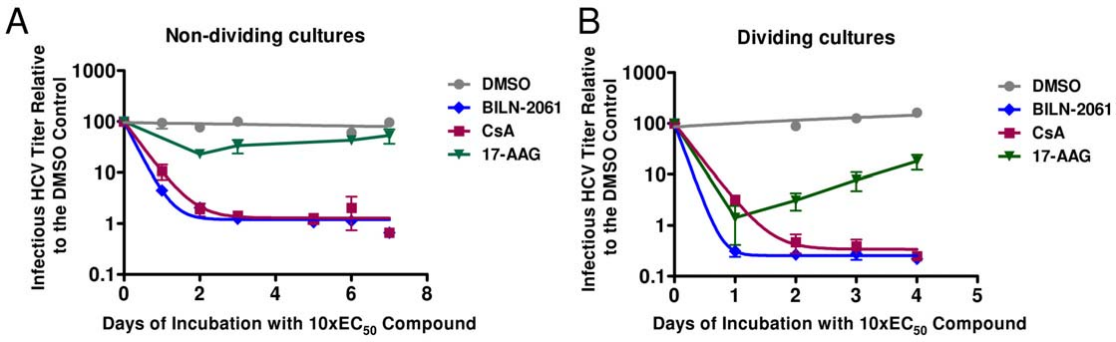
17-AAG reduces HCV titer by slowing cellular growth. (A) Non-dividing HCV-infected cultures were treated with 10×EC_50_ for each compound. Subsequently, extracellular HCV titer was quantified at various times in the presence of DMSO (circles), 0.35 µM HSP90 inhibitor 17-AAG (triangles), 1.30 µM cyclosporin inhibitor CsA (squares), or 1.40 µM HCV protease inhibitor BILN-2061 (diamonds). The DMSO data were fit to a linear equation. The CsA and BILN-2061 data were fit to second-order exponential equations. The 17-AAG data could not be fit to first or second order exponential equations. (B) Dividing HCV-infected cultures were treated with 10×EC_50_ compound. Subsequently, extracellular HCV titer was quantified at various times in the presence of DMSO (circles), 0.35 µM HSP90 inhibitor 17-AAG (triangles), 1.30 µM cyclophilin inhibitor CsA (squares), or 1.40 µM HCV protease inhibitor BILN-2061 (diamonds). The DMSO data were fit to a linear equation. The CsA and BILN-2061 data were fit to second-order exponential equations. The 17-AAG data could not be fit to first or second order exponential equations.

Because 17-AAG slows cellular proliferation at concentrations similar to its effective concentration for inhibiting HCV replication, we next assessed whether it inhibited HCV replication in non-dividing cells. We first established a non-dividing monolayer of Lunet-CD81 cells [24] and then infected that monolayer with cell-culture adapted virus capable of spreading and infecting all of the cells in a culture [25]. After the cell cultures were ∼100% infected, we added compounds at a concentration 10-fold above the EC_50_ (Fig. S1) and measured the extracellular viral titer produced at several time points over a 7-day incubation (see Materials and Methods). When the fully-infected non-dividing cultures were treated with DMSO alone, the extracellular viral titer remained relatively constant at between 10^5^ and 10^6^ TCID_50_/ml over time (Fig. 6A). However, treatment with 10-fold excess (*i.e.* 10×EC_50_) CsA or HCV protease inhibitor BILN-2061 resulted in a sustained 2-log suppression of infectious HCV titer over the 7-day time course (Fig. 6A). In contrast, 17-AAG treatment resulted in a much more modest (0.6 log on day 2) and transient (0.3 log on Day 7) reduction of infectious HCV titer (Fig. 6A). The reduction in HCV titer caused by 17-AAG, may at least in part, result from the modest toxicity observed in the non-dividing monolayers at the concentrations used. 17-AAG treatment reduced the number of cells to 74% of the DMSO-treated control, but neither CsA nor BILN 2061-treatment decreased the number of cells in the non-dividing monolayers (data not shown).

As a comparative control, we performed the same compound-treatment using fully-infected cultures that were still dividing (see Materials and Methods). 17-AAG treatment reduced the level of infectious HCV in dividing cells by roughly 2 logs after 24 hours of treatment (Fig. 6B). The inhibitory effect of 17-AAG was diminished only as the cell cultures reached confluence (days 3 and 4 post-compound addition) (Fig. 6B). These results suggest that HCV replication inhibition by 17-AAG is caused by suppression of cell proliferation. In contrast, CsA and BILN-2061 inhibited viral titers in both dividing and non-dividing cells.

## Discussion

Targeting host factors essential for viral replication has the potential to identify new antiviral molecules with high barriers to drug resistance. Toward this end, many groups have screened for host factors essential for viral replication [26]–[28]. However, this strategy requires thorough assessment of cellular toxicity to ensure that an observed antiviral activity is not confounded by inhibition of cellular proliferation. From the data reported here, we concluded that effects on cell growth correlate with the antiviral activity observed for molecules like gemcitabine and 17-AAG. We also found that certain cell-based assays using biochemical surrogates for cell number may under-represent the effects of these molecules. However, direct measurements of cell number and cell growth clearly distinguished selective antivirals, like CsA, from molecules that slow cellular proliferation.

The term “toxicity” is broadly applied to adverse effects on cellular viability, and our observations underscore the complexity of accurately measuring toxicity. Compounds such as puromycin exert a toxicity that is rapid, apoptotic, and routinely-detectable. The toxicity of 17-AAG, in contrast, is distinct both in mechanism and cellular phenotype. While puromycin blocks protein synthesis and results in rapid cellular apoptosis, 17-AAG inhibits HSP90 and reduces cellular viability without inducing rapid apoptosis. Phenotypically, the magnitude of 17-AAG toxicity was comparable to puromycin in the intracellular ATP and trypan-blue exclusion assays. The intracellular esterase activity assay, in contrast, was not as sensitive a measure of 17-AAG toxicity. Perhaps intracellular esterases are not affected, or may even accumulate, as 17-AAG slows cell growth. Direct measurements of cellular confluence suggested that 17-AAG was reducing the overall rate of cell growth. This indicates that 17-AAG's effects are neither immediate (see 20–30 hour timepoints in Figure 5) nor lytic (Figure 4). Because viral replication may be inhibited by subtle changes in cellular growth and viability, it is essential to fully characterize the “toxicity” of a small-molecule inhibitor before concluding its antiviral activity is truly selective.

Toxicity assessments at single time points are insufficient to characterize a compound's effect on cellular growth rate. Kinetic measurements of cellular proliferation were essential to distinguish delayed toxicity (in which cells grow at normal rates for 2 days then begin to lyse between days 2 and 3) from an alteration of cellular growth rates for 17-AAG. These observations emphasize the value of a comprehensive assessment of cell viability and proliferation in antiviral drug discovery.

A potential limitation of many *in vitro* systems used to assess antiviral potency, such as the HCV replicon cell line described in this report, is that they rely on rapidly-dividing immortalized cells as viral hosts. When evaluating the antiviral potential of host factors, this creates two distinct problems. First, alterations in cellular growth rates may be responsible for the perceived antiviral effects (as we observed for puromycin and 17-AAG). For the HCV replicon, this is due to the dependence on cellular division. Shortly after the discovery of the HCV replicon, cellular confluence was observed to significantly reduce replicon copy number [18], [29]. Second, the intracellular environment in immortalized cells is fundamentally different than that in the primary cells that HCV infects *in vivo*. For example, HSP90 is known to reside in complexes with co-chaperones that are distinct for primary or immortalized cells [30].

While 17-AAG did not selectively inhibit HCV in our *in vitro* assays, the activity observed for other viruses may be more promising. The mechanisms by which HSP90 promotes viral replication seem to be distinct between viruses [3], [7], [8], [11], [16]. Some mechanisms may be independent from the effect on cellular growth rate responsible for the anti-HCV activity. In the case of poliovirus, HSP90 inhibition limits the number of competent viruses produced from primary cells and specifically interferes with capsid maturation [3]. Underscoring the differences in susceptibility between viruses, HSP90 inhibitor monotherapy reduced viral load of poliovirus-infected mice [3] but not HCV-infected mice [31]. Furthermore, for acute indications where the duration of viral infection is limited (*i.e.*, influenza or rhinovirus), mild effects on host cell proliferation may be tolerated. However, in standard HCV replicon cells the apparent antiviral activity of 17-AAG is dependent on its ability to slow the cellular replication rate whereas the antiviral activity of CsA was not. Thus, for HCV therapy CypA appears to be a more promising target than HSP90.

## Materials and Methods

### Cell Culture

51C-1a-TdTomato [32], 51C-H77-Rluc [33], and Lunet-CD81 [25] cell lines were propagated in Dulbecco's Modified Eagle Medium (D-MEM) with GlutaMAX™-I (Invitrogen, Carlsbad, CA) supplemented with 10% FBS (HyClone, Logan, UT), 1 unit/ml Penicillin (Invitrogen), 1 µg/ml Streptomycin (Invitrogen), and 0.1 mM non-essential amino acids (Invitrogen). Replicon cell lines were selected and maintained in 0.5 mg/ml G-418 (Invitrogen).

### Antiviral compounds

BILN-2061 was purchased from Acme Bioscience (Belmont, CA). HCV-796 was synthesized by Curragh Chemistries (Cleveland, OH). 17-AAG was purchased from A. G. Scientific (San Diego, CA). Cyclosporin A was purchased from Alexis Biochemicals (San Diego, CA). Colchicine was purchased from MicroSource (Gaylordsville, CT). Gemcitabine was purchased from Toronoto Research Chemicals (North York, Ontario, Canada). Puromycin was purchased from Sigma Aldrich (St. Louis, MO).

### Antiviral assays

Renilla luciferase replicon cell assays were performed as previously described [33]. Calcein AM (Anaspec, Fremont, CA) and CellTiter-Glo (Promega, Madison, WI) readouts were performed as instructed by the manufacturer.

For direct assessment of cell number, genotype 1a replicon cells were seeded in 384 well plates at 1500 cells/well. Compounds were added as previously described [33]. Cells were fixed in 1% paraformaldehyde (Electron Microscopy Sciences) and 25 µg/ml Hoechst 33342 (Invitrogen) in PBS for 30 minutes at room temperature. The fixed cells were washed twice with PBS, and images of each well were acquired with the ImageXpress Micro (Molecular Devices, Sunnyvale, CA). Nuclei were quantified with the cell scoring application in MetaXpress (Molecular Devices, Sunnyvale, CA).

To assess cellular growth, 96 well plates ImageLock plates (Essen Instruments, Ann Arbor, MI) were seeded at a density of 5×10^3^ cells/well in culture medium without G418. Percent confluence was quantified using bright phase images (at 10× magnification) acquired of each well every four hours in an IncuCyte (Essen Instuments, Ann Arbor, MI). Alternatively, 7.5×10^4^ cells/well were plated in 6 well plates. Each day post drug addition, cells were dislodged from the tissue culture plate using 350 µL trypsin-EDTA and then quenched with 150 µL fetal bovine serum. The number of cells that exclude trypan-blue was determined using the Vi-Cell (Beckman Coulter, Fullerton, CA) in triplicate.

To assess the antiviral potency of compounds in rapidly-dividing virus-infected cells. we adapted a method from a previously described assay [34]. Briefly, 1500 cells per well were plated in 384 well-plates. Infection with the HCVcc Min3 virus (core K78E, NS2 W879R, and NS4B K1761N) [25] was followed by addition of compound as described [33]. After three days, intracellular NS3-4A protease activity was assessed [34]. Luciferase, Calcein, CellTiter-Glo, and NS3-4A levels were expressed as a percent of the no-drug control.

### HCV-infected non-dividing cultures

HCV-infected non-dividing cultures were established by methods adapted from Sainz and Chisari [24]. Briefly, Lunet-CD81 cells [25] were seeded in 12-well plates at a density of 50,000 cells/well. The plates were incubated over-night at 37°C and subsequently DMSO was added to 1% final concentration. Cells were allowed to grow to confluence over three days. Infectious HCV (2a) with three adaptive mutations (the Min3 virus) (core K78E, NS2 W879R, and NS4B K1761N) [25] was then added to each well at an MOI of five. Infection was permitted to spread for seven days until the cultures were ∼100% infected (by NS5A immunofluorescence). Subsequently, compounds of interest were added at 10×EC_50_ concentration. 200 µl aliquots of the extracellular medium were saved daily, stored at −80°C, and subsequently used for infectious viral titering. After three days, the compound-medium was refreshed and time points were collected for seven days. Viral titers as a percentage of the DMSO-treated control were measured through diluting the extracellular medium 40-fold and using it to infect naïve Lunet-CD81 cells. After 72 hours of incubation, intracellular HCV replication was measured by following NS3-4A protease cleavage of a europium-labeled peptide [35].

As a comparative control, fully-infected, growing cell cultures were prepared as well. Briefly, cells were plated at 25,000 cells/well on 12-well plates. After the cells adhered to the plate (four hours post-seeding), they were infected at an MOI of five using adapted HCV (2a) (see above). The HCV infection was permitted to spread until the cultures were fully infected (three days post-infection). Subsequently, compounds were added and extracellular medium was removed each day and analyzed as described above.

## Supporting Information

Figure S1 **CsA and 17-AAG maintain potency, similar to rapidly-dividing replicon cells, in rapidly-dividing virus-infected cells.** The normalized amount of virus, as measured by enzymatic activity of the NS3-4A protease, was determined as a function of dose. (A) CsA EC_50_ was 130 nM (259 nM against the replicon), and (B) 17-AAG EC_50_ was 35 nM (12.1 nM against the replicon). (C) As a control, the protease inhibitor BILN-2061 exhibited an EC_50_ of 140 nM.
(TIF)

## References

[1] Reichard O, Schvarcz R, Weiland O (1997). Therapy of hepatitis C: alpha interferon and ribavirin.. Hepatology.

[2] Rong L, Dahari H, Ribeiro RM, Perelson AS (2010). Rapid emergence of protease inhibitor resistance in hepatitis C virus.. Sci Transl Med.

[3] Geller R, Vignuzzi M, Andino R, Frydman J (2007). Evolutionary constraints on chaperone-mediated folding provide an antiviral approach refractory to development of drug resistance.. Genes Dev.

[4] Patel H, Heathcote EJ (2010). Sustained virological response with 29 days of Debio 025 monotherapy in hepatitis C virus genotype 3.. Gut.

[5] Le Pogam S, Seshaadri A, Ewing A, Kang H, Kosaka A (2010). RG7128 alone or in combination with pegylated interferon-alpha2a and ribavirin prevents hepatitis C virus (HCV) Replication and selection of resistant variants in HCV-infected patients.. J Infect Dis.

[6] Ujino S, Yamaguchi S, Shimotohno K, Takaku H (2010). Combination therapy for hepatitis C virus with heat-shock protein 90 inhibitor 17-AAG and proteasome inhibitor MG132.. Antivir Chem Chemother.

[7] Ujino S, Yamaguchi S, Shimotohno K, Takaku H (2009). Heat-shock protein 90 is essential for stabilization of the hepatitis C virus nonstructural protein NS3.. J Biol Chem.

[8] Smith DR, McCarthy S, Chrovian A, Olinger G, Stossel A (2010). Inhibition of heat-shock protein 90 reduces Ebola virus replication.. Antiviral Res.

[9] Hu J, Toft DO, Seeger C (1997). Hepadnavirus assembly and reverse transcription require a multi-component chaperone complex which is incorporated into nucleocapsids.. Embo J.

[10] Hu J, Seeger C (1996). Hsp90 is required for the activity of a hepatitis B virus reverse transcriptase.. Proc Natl Acad Sci U S A.

[11] Chase G, Deng T, Fodor E, Leung BW, Mayer D (2008). Hsp90 inhibitors reduce influenza virus replication in cell culture.. Virology.

[12] Modi S, Stopeck AT, Gordon MS, Mendelson D, Solit DB (2007). Combination of trastuzumab and tanespimycin (17-AAG, KOS-953) is safe and active in trastuzumab-refractory HER-2 overexpressing breast cancer: a phase I dose-escalation study.. J Clin Oncol.

[13] Sausville EA, Tomaszewski JE, Ivy P (2003). Clinical development of 17-allylamino, 17-demethoxygeldanamycin.. Curr Cancer Drug Targets.

[14] Fraser JS, Clarkson MW, Degnan SC, Erion R, Kern D (2009). Hidden alternative structures of proline isomerase essential for catalysis.. Nature.

[15] Prodromou C, Pearl LH (2003). Structure and functional relationships of Hsp90.. Curr Cancer Drug Targets.

[16] Okamoto T, Nishimura Y, Ichimura T, Suzuki K, Miyamura T (2006). Hepatitis C virus RNA replication is regulated by FKBP8 and Hsp90.. Embo J.

[17] Yang F, Robotham JM, Grise H, Frausto S, Madan V (2010). A major determinant of cyclophilin dependence and cyclosporine susceptibility of hepatitis C virus identified by a genetic approach.. PLoS Pathog.

[18] Nelson HB, Tang H (2006). Effect of cell growth on hepatitis C virus (HCV) replication and a mechanism of cell confluence-based inhibition of HCV RNA and protein expression.. J Virol.

[19] Stuyver LJ, McBrayer TR, Tharnish PM, Hassan AE, Chu CK (2003). Dynamics of subgenomic hepatitis C virus replicon RNA levels in Huh-7 cells after exposure to nucleoside antimetabolites.. J Virol.

[20] Kneteman NM, Howe AY, Gao T, Lewis J, Pevear D (2009). HCV796: A selective nonstructural protein 5B polymerase inhibitor with potent anti-hepatitis C virus activity in vitro, in mice with chimeric human livers, and in humans infected with hepatitis C virus.. Hepatology.

[21] Allen DW, Zamecnik PC (1962). The effect of puromycin on rabbit reticulocyte ribosomes.. Biochim Biophys Acta.

[22] Handschumacher RE, Harding MW, Rice J, Drugge RJ, Speicher DW (1984). Cyclophilin: a specific cytosolic binding protein for cyclosporin A.. Science.

[23] Malawista SE, Bensch KG (1967). Human polymorphonuclear leukocytes: demonstration of microtubules and effect of colchicine.. Science.

[24] Sainz B, Chisari FV (2006). Production of infectious hepatitis C virus by well-differentiated, growth-arrested human hepatoma-derived cells.. J Virol.

[25] Pokrovskii MV, Bush CO, Beran RK, Robinson MF, Cheng G (2010). Novel mutations in a tissue-culture adapted HCV strain improve infectious virus stability and markedly enhance infection kinetics.. J Virol.

[26] Flajolet M, Rotondo G, Daviet L, Bergametti F, Inchauspe G (2000). A genomic approach of the hepatitis C virus generates a protein interaction map.. Gene.

[27] Tai AW, Benita Y, Peng LF, Kim SS, Sakamoto N (2009). A functional genomic screen identifies cellular cofactors of hepatitis C virus replication.. Cell Host Microbe.

[28] Milad M, Sullivan W, Diehl E, Altmann M, Nordeen S (1995). Interaction of the progesterone receptor with binding proteins for FK506 and cyclosporin A.. Mol Endocrinol.

[29] Pietschmann T, Lohmann V, Rutter G, Kurpanek K, Bartenschlager R (2001). Characterization of cell lines carrying self-replicating hepatitis C virus RNAs.. J Virol.

[30] Kamal A, Thao L, Sensintaffar J, Zhang L, Boehm MF (2003). A high-affinity conformation of Hsp90 confers tumour selectivity on Hsp90 inhibitors.. Nature.

[31] Nakagawa S, Umehara T, Matsuda C, Kuge S, Sudoh M (2007). Hsp90 inhibitors suppress HCV replication in replicon cells and humanized liver mice.. Biochem Biophys Res Commun.

[32] Robinson M, Tian Y, Delaney WEt, Greenstein AE (2011). Preexisting drug-resistance mutations reveal unique barriers to resistance for distinct antivirals.. Proc Natl Acad Sci U S A.

[33] Robinson M, Yang H, Sun SC, Peng B, Tian Y (2010). Novel HCV Reporter Replicon Cell Lines Enable Efficient Antiviral Screening against Genotype 1a.. Antimicrob Agents Chemother.

[34] Cheng G, Chan K, Yang H, Corsa A, Pokrovskii M (2011). Selection of Clinically-relevant Protease Inhibitor Resistant Viruses Using the Genotype 2a HCV Infection System.. Antimicrob Agents Chemother.

[35] Paulson MS, Yang H, Shih IH, Feng JY, Mabery EM (2009). Comparison of HCV NS3 protease and NS5B polymerase inhibitor activity in 1a, 1b and 2a replicons and 2a infectious virus.. Antiviral Res.

