# Supplementary figures and images for: Cellular Growth Kinetics Distinguish a Cyclophilin Inhibitor from an HSP90 Inhibitor as a Selective Inhibitor of Hepatitis C Virus
Source: PLoS One. 2012 Feb 8;7(2):e30286. doi: 10.1371/journal.pone.0030286 (PMC3275588; doi:10.1371/journal.pone.0030286)

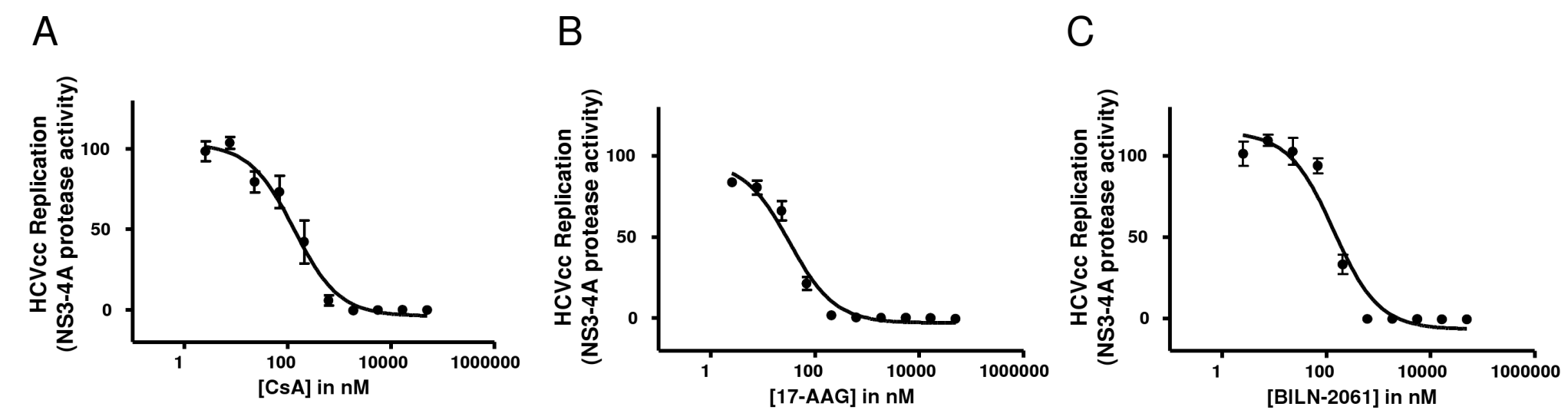

Supplement: Figure S1 — CsA and 17-AAG maintain potency, similar to rapidly-dividing replicon cells, in rapidly-dividing virus-infected cells. The normalized amount of virus, as measured by enzymatic activity of the NS3-4A protease, was determined as a function of dose. (A) CsA EC50 was 130 nM (259 nM against the replicon), and (B) 17-AAG EC50 was 35 nM (12.1 nM against the replicon). (C) As a control, the protease inhibitor BILN-2061 exhibited an EC50 of 140 nM. (TIF) [file pone.0030286.s001.tif]
